# Opto-electrical evaluation of visible blind fast-response nanostructured SnO_2_/Si photodetector†

**DOI:** 10.1039/d4ra05303f

**Published:** 2024-09-02

**Authors:** Ethar Yahya Salih

**Affiliations:** a College of Energy and Environmental Sciences, Al-Karkh University of Science 10081 Baghdad Iraq ethar988@gmail.com ethar@kus.edu.iq

## Abstract

In this study, a nanostructured tin(iv) oxide (SnO_2_)/Si heterojunction UV photodetector was fabricated in response to laser pulses attained *via* pulsed laser deposition (PLD). In particular, the photo-detection mechanisms of the proposed devices were thoroughly investigated considering multiple-profile dependency, namely, laser pulses, spectral response, and incident power. In detail, particle diameters of 25 and 41 nm with a bandgap alteration of 0.2 eV and a cut-off phenomenon at around 335 nm occurred as a result of an increase in the number of pulses from 300 to 700. The optimum photodetector (at 700 pulses, *λ*_340_ nm, and 10 mW cm^−2^) revealed a responsivity (*R*_*λ*_) and external quantum efficiency (EQE) of 32.9 mA W^−1^ and 120.2, respectively. Furthermore, a descended photocurrent behavior from 330 to 63.9 (μA) was observed at wavelengths of 340 and 625 nm with a visible light rejection ratio of 516%, indicating the visible blind characteristic of the proposed geometry. This was also observed at an extremely low bias potential (0.01 V). The incident power profile demonstrated an inversely proportional correlation to *R*_*λ*_ and EQE, with values 37.8 mA W^−1^ and 137.7 at 6 mW cm^−2^, respectively. Of the fabricated devices, the photodetector performance attained at 700 pulses, *λ*_340_ nm, and 10 mW cm^−2^ depicted a substantially rapid time-resolved characteristic with a rise and fall time of 0.29 and 0.31 s, respectively.

## Abstract 2

Considerably high photoresponsive characteristics of an extremely low bias driven, fast-response, and visible-blind SnO_2_/Si heterojunction photodetector *via* pulsed laser deposition.

## Graphical abstract

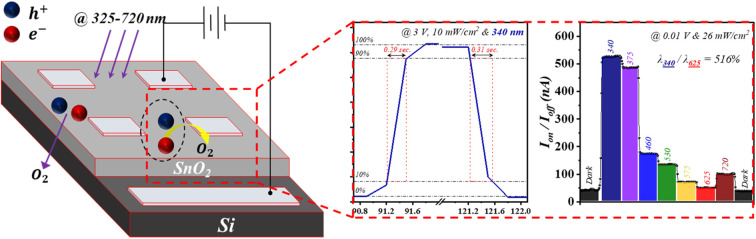

## 1. Introduction

Optoelectronics-based porous silicon (Si) technology is being broadly investigated as a substitute for the outdated bulk Si pathway. The former is widely considered within research and manufacturing fields because of its wide-reaching applications to meet the evolving demands, for instance, military UV/vis/IR communication and monitoring, flame detection, environmental and biological research, and traditional imaging. Compared to traditional bulk Si, porous Si has the advantages of easy processing, cost-effectiveness, and enhanced opto-electrical functionality; moreover, porosification allows for a cutting-edge performance under ambient settings.^1–4^ However, technology-based porous silicon for optoelectronics, in particular terms “UV photodetectors”, necessitates subsequent development within the confines of significant visible-spectrum rejection, sound time-resolved characteristics, efficiency, high UV sensitivity, low driving force consumption, *etc.* Specifically, there are two main photodetection mechanisms: photoconductive and photovoltaic effects, and the latter accounts for p–n/p–i–n homo-/heterojunctions and Schottky barrier photodetectors.^5,6^ To further enhance the aforementioned UV photodetection mechanisms in both photoconductive and photovoltaic effects, researchers are increasingly focused on advancing the properties of porous Si photodetectors. In conjunction, a variety of nanostructured photodetector-based porous Si materials are being given due consideration like zinc oxide (ZnO), cadmium sulfide (CdS), nickel-oxide (NiO), copper oxide (CuO), and cadmium oxide (CdO);^7–12^ most of which operate at high driving force, along with a relatively low time-resolved characteristic, especially in the UV region. This could restrict the self-reliance of application.^13,14^ SnO_2_ is a widely acknowledged n-type semiconductor with a considerably wide optical band gap (≈3.5–3.7 eV) at room temperature. Such a wide band gap allows this type of metal oxide to be an almost perfect UV absorber with a substantial visible spectrum rejection, by which a number of target applications can be demonstrated.^15–17^ Specifically, the addressed semiconductor demonstrated substantial optoelectronic behavior in several occurrences. For example, SnO_2_ demonstrated a considerable figure of merits with a high rectification ratio within the heterojunction photodetector framework^18–20^ and also as a heterojunction solar cell, especially upon doping with other semiconductors, such as ZnO.^21^

In this context, this study demonstrates a systematic illustration concerning the opto-electrical and photodetection mechanisms of the n-SnO_2_/p-Si visible blind UV photodetector with an exceedingly fast time-resolved characteristic. Furthermore, the effect of laser pulses utilized during SnO_2_ deposition is elucidated. In particular, the optimum device, fabricated using 700 pulses, revealed a rise/fall time of 0.29 and 0.31 s, respectively. A direct correlation between the structural features of the SnO_2_ layer along the final device opto-electrical performance is also well-oriented.

## 2. Experimental procedure

### 2.1 Metal oxide layer deposition

In a typical solution process procedure, SnO_2_ nanoparticles were prepared through a continuous stirring of SnCl_2_·H_2_O (0.15 M) in 100 mL of deionized water until a total homogenous solution was acquired. NaOH (1.25 M) was added dropwise to the solution for high quality crystal growth until a pH of 9 was obtained. Meanwhile, the acquired solution was autoclaved at 65 °C continuously for 3 h. The obtained product was then subjected to a multi-cycle washing procedure using EtOH for the removal of undesired organics residuals. The latter was dried at 75 °C, and later thermally treated for 5 h. The resulting powder was pressed under a 5 ton pressure to produce a deposition target that was 1 cm in diameter and 2 mm in thickness.

### 2.2 Device fabrication

Si substrate (p-type, ∼523 μm, ∼2−11 Ω) porosification was attained *via* electrochemical etching procedure in a Teflon cell. In detail, a pre-cleaned Si wafer was placed inside the PTFE cell containing a mixture of EtOH and hydrofluoric acid with a ratio of 1 : 4 (v/v). Next, a current density of 35 mA cm^−2^ was applied between the utilized electrodes, Si-anode and Pt-cathode, under halogen illumination of 250 W for 15 min. For the deposition of the SnO_2_ layer, the fabricated target was positioned vertically inside a PLD chamber with a distance of 2 cm to the Si wafer. The positioned target was then irradiated at 45° using a second harmonic Nd : YAG laser with an energy of 300 mJ, while the number of pulses was varied from 300 to 700; the wavelength, repetition rate, and frequency utilized during PLD procedure were fixed to 532 nm, 10 ns, and 6 Hz, respectively. The attained layers, with respect to the utilized number of pluses, were thermally treated at 400 °C for 1 h with a heating/cooling rate of 5 °C min^−1^. For a complete device geometry, the Ag metal contact, inset in Fig. 3(a and b), was thermally evaporated on both p-Si and n-SnO_2_ layers using mechanical and diffusion pumps (10^−5^ mbar).

### 2.3 Characterization and device measurement

The SnO_2_ structural features were characterized *via* X-ray diffractions technique (Rigak D-MAX-2200, XRD), as well as Raman spectroscopy (WITec), whereas the optical behavior was inspected through ultraviolet-visible light spectroscopy (DiNovix, UV-vis). In conjunction, the photoluminescence (PL, PerkinElmer LS-50B) analysis was performed at 325 nm as the excitation wavelength using a xenon light source. The deposited layer topographies were determined through field emission-scanning electron microscopy (FE-SEM, FEI Quanta-400). The opto-electrical characteristics of the fabricated photodetectors were tested under dark and illumination conditions using a Keithley 2401 SMU instrument, in conjunction with multi-wavelength narrow bandpass filters (ThorLabs). This particular measurement was repeatedly performed over 5 cycles, for which the mean value at particular supplied bias was presented. This includes both 3 and 0.01 bias voltages, at which the figure of merits were measured, while the time-resolved characteristic was investigated considering multiple cycles at continuous altered illumination intensities, along with a linear search from 10% to 90%.

## 3. Results and discussion

### 3.1 Deposited layer characteristics

**Fig. 1:**
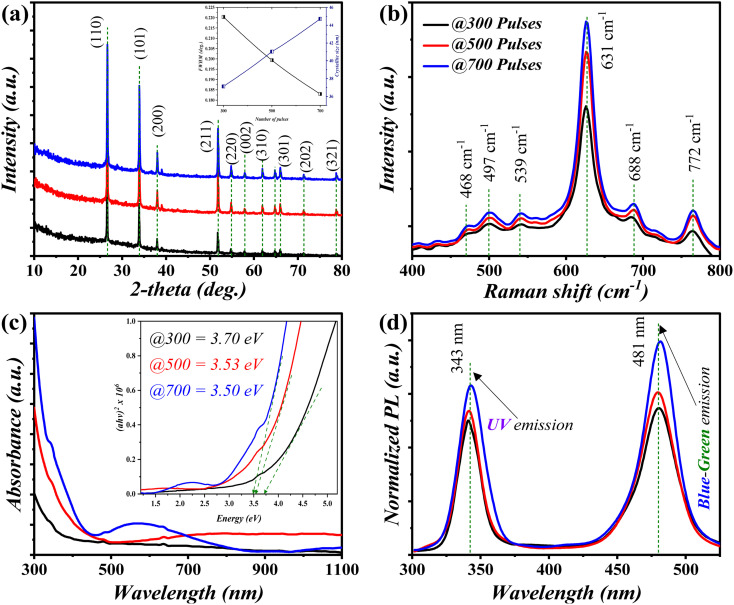
(a) XRD patterns, (b) Raman spectra, (c) absorption spectra, and (d) PL spectra of the fabricated SnO_2_ as a function of the number of pulses.

Fig. 1(a) demonstrates the XRD pattern of the deposited SnO_2_ nanostructured layer/s as a function of utilized number of pulses through which a well-crystallized tetragonal rutile structure (JCPDS no. 01-0657) was attained. No additional impurities were observed in the XRD spectra. However, it could also clearly be perceived from the XRD patterns that increasing the number of pulses resulted in higher crystallinity of the attained SnO_2_ layer. This was further investigated *via* the FWHM and the calculated crystallite size using the Debye Scherrer equation,^22^ inset in Fig. 1(a). In detail, a higher crystal quality indicator (FWHM) was acquired with a higher number of pulses. This was inversely noted for the crystallite size profile, wherein the crystallite sizes of 37.2, 41.0, and 44.7 nm were obtained for the SnO_2_ nanostructured layers acquired using 300, 500, and 700 pulses, respectively. The investigated Raman shift (Fig. 1(b)) exhibited three main peaks positioned at around 468, 631, and 772 cm^−1^, which corresponded to the rutile SnO_2_ modes, E_g_, A_g_, and B_2g_,^23^ respectively. The mode attained at 468 cm^−1^ is relatively small for all discussed layers, which indicates the occurrence of an oxygen vacancy (V_O_) within the deposited layers. The additional two weak peaks acquired at around 497 and 688 cm^−1^ are related to the A_2u_ (TO) and A_2u_ (LO) modes, respectively. The peak attained at 539 cm^−1^ corresponds to the Raman forbidden mode (B_iu_).^24,25^ The optical properties of the acquired layer/s (Fig. 1(c)) indicated a strong cut-off singularity at around 335 nm, along with a noticeable hyperchromic shift as the number of pulses increased. Furthermore, the optical band gap, inset in Fig. 1(c), was estimated using the Tauc relation.^26^ Particularly, the SnO_2_ layers deposited using 300, 500, and 700 laser pulses exhibited band gap values of 3.7, 3.53, as well as 3.5 eV, respectively. In conjunction, the PL emission (Fig. 1(d)) demonstrates two extensive peaks at around 343 and 481 nm, which corresponded to the UV and blue-green emissions, respectively. The former could be ascribed to the near transmission band-edge resulting from the recombination of holes and electrons in CB and VB, respectively. However, the latter is mainly due to the defect levels of electron transition in the BG, which in turn, is attributed to oxygen vacancies on the deposited SnO_2_ surface.^27^

**Fig. 2:**
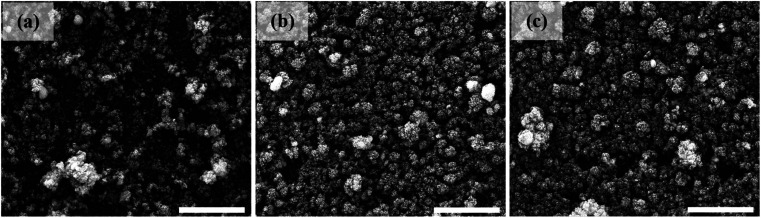
FE-SEM features of the deposited SnO_2_ layer/s using (a) 300, (b) 500, and (c) 700 laser pulses (scale bar = 1 μm).

Fig. 2 depicts the surface features of the deposited nanostructured SnO_2_, in which mostly compact and uniform nanoparticle distribution features can be perceived. In conjunction, the average diameter/s of the attained nanoparticles for the utilized laser energies exhibited a decreasing trend as a function of the increasing laser energy from 140 to 220 mJ. In detail, nanoparticle diameters of 25, 32, and 41 nm were observed for laser energies of 140, 180, and 220 mJ, respectively. In addition, the utilized laser energy increment resulted in higher density deposited layers. The FE-SEM profile delivered an inverse proportionality to that attained in the UV-vis analysis, as shown in the inset of Fig. 1(c).

### 3.2 Opto-electronic performance evaluation of the device

#### 3.2.1 Laser pulses profile

**Fig. 3:**
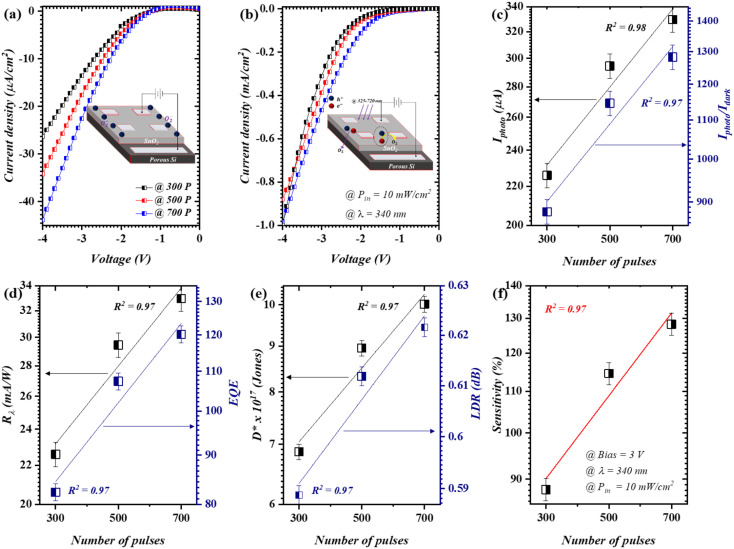
Opto-electrical features of devices by means of laser pulses: (a) dark *I*–*V*, (b) illuminated *I*–*V*, (c) *I*_photo_ and *I*_photo_/*I*_dark_ ratio, (d) *R*_*λ*_ and EQE, (e) D* and LDR, and (f) *S* %; figure of merits were attained at 3 V, 340 nm, and 10 mW cm^−2^.

Fig. 3(a and b) elucidates the reversed bias *I*–*V* characteristics of the photodetectors under dark and illuminated (*P*_in_ = 10 mW cm^−2^) states, respectively. The linear forward and reverse *I*–*V* are provided in the ESI (Fig. S1†). The substantial current enhancement (Fig. 3(c)) provoked *via* photo-excited electron–hole pair generation was found to be around 1400% (*I*photo/*I*dark) under 340 nm UV illumination for the photodetector obtained using 700 pulses. It is widely recognized that for the n-type layer (SnO_2_), a reversed saturation current under a dark state is generated as a result of oxygen molecule adsorption. Thereupon, an electron is captured from the SnO_2_ layer. Subsequently, a depletion region is formed [O_2_(g) + e^−^ → O^−^_2_(ad)], as shown in the inset of Fig. 3(a). Upon 340 nm UV illumination with a high energy photon (≥3.5 eV), the photo-generated hole shifts to the surface and reunifies with the pre-adsorbed electron [h^+^ + O^−^_2_(ad) → O_2_(*g*)], as shown in the inset of Fig. 3(b). As a consequence, a substantial photo-generated reversed current could be observed.^28^ The reversed bias *I*–*V* profile corresponded almost linearly to the increase in the number of pulses. Such phenomenon could be due to the fact that smaller nanoparticles are formed at a low number of pulses. The ablated duration increased as the number of laser pulses increased, which resulted in the agglomeration of nanoparticles. This in turn leads to a higher conductivity at a higher number of pulses, as demonstrated in the *I*–*V* profile. The described behavior could be clearly perceived in Fig. 3(c), through which an unmitigated linear correlation of the illumination current (*I*_photo_) was obtained (*R*^2^ = 0.98). This was attained using a linear fit (independent fit), where *R*^2^ characterizes a measure of the fitting model quality on the data.

In particular, the presented figure of merits (Fig. 3(c)) were measured over multiple times, while only the mean value was demonstrated. Furthermore, the proposed geometry was also tested considering the related figure of merits; these include the responsivity [*I*_photo_/*P*_in_], effective quantum efficiency [(*I*_photo_/*e*)(*P*_in_/*hv*)], detectivity [D* = *R*_*λ*_*A*/(2*qI*_dark_)^1/2^], and linear dynamic range [LDR = 20log (I_photo_/I_dark_)]. Herein, *q* is the electron charge and *A* is the working area of a photodetector.^29^ As a function of the laser pulse count, the stated figure of merits exhibited a linear correlation (*R*^2^ ≥ 0.97) with increasing number of pulses. This can be clearly seen in Fig. 3(d through f). In particular, the optimum device (@700) produced *R*_*λ*_ and EQE values of 32.9 mA W^−1^ and 120.2, respectively. The attained EQE values were observed to be superior to unity at high number of pulses (pulses ≥500), which indicate the elevated light trapping ability of the fabricated photodetectors.

#### 3.2.2 Spectral response profile

**Fig. 4:**
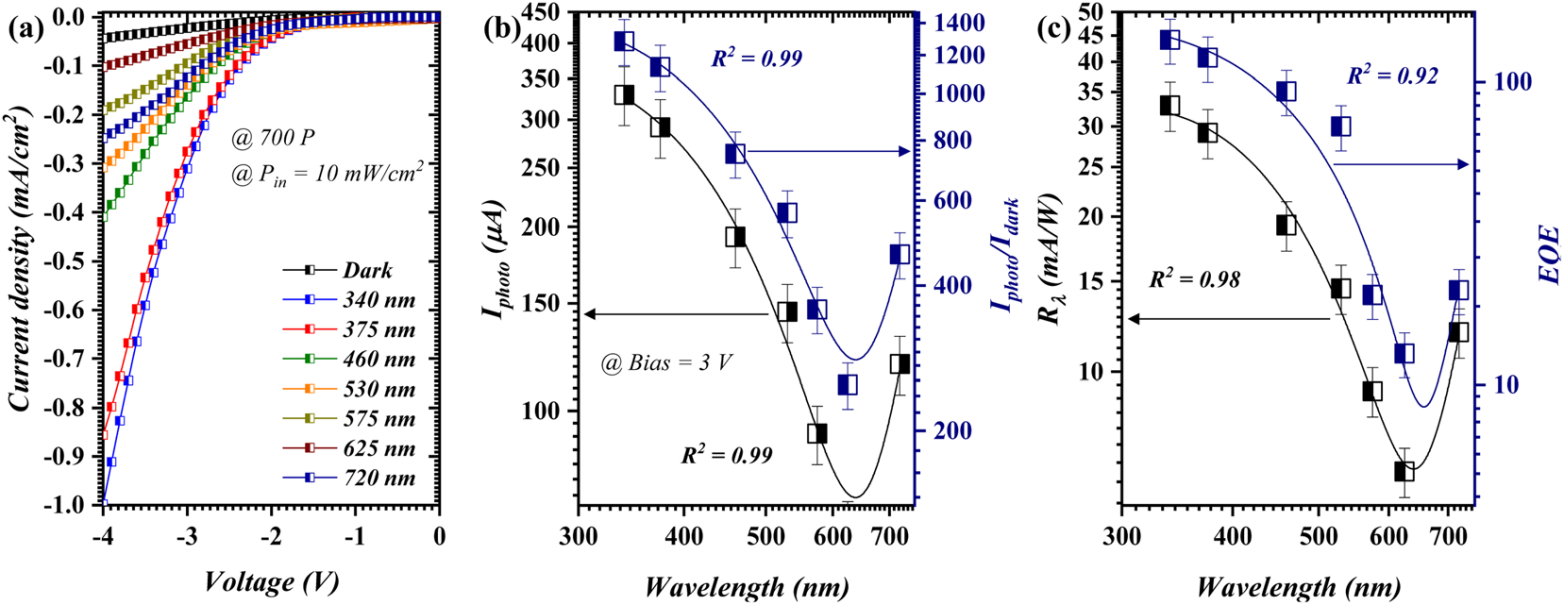
Opto-electrical features of the fabricated photodetector at 700 pulses as a function of wavelength; (a) *I*–*V* profile, (b) *I*_photo_ and *I*_photo_/*I*_dark_ ratio, and (c) *R*_*λ*_ and EQE.

Fig. 4 elucidates the spectral (*λ* = 340 − 720 nm) response profile of the fabricated device using 700 pulses. The reversed *I*–*V* characteristics (Fig. 4(a)) typified a distinctive decrease as the scanned wavelength increased from 340 to 625 nm. The demonstrated *I*–*V* characteristics revealed a logically visible-blind behavior in the addressed geometry. Such an event can be further distinguished in the Iphoto profile (Fig. 4(b), – navy curve), wherein the captured *I*photo decreased from 330 to 63.9 (μA) through the specified wavelengths, respectively, and a visible light rejection ratio (*λ*_340_/*λ*_625_) of 516%. The *R*^2^ value was determined to be 0.99 for both investigated profiles. This was attained using a cubic polynomial fitting function, which describes the highest degrees of a fit. Particularly, *R*^2^ delivers evidence about the goodness of fit of a model (±1). The stated episode was even further validated through the *R*_*λ*_ curve (Fig. 4(c)), for which a similar Iphoto observation was acquired at 10 mW cm^−2^. Such a singularity could be mainly because of the fact that the generated photoinduced carriers responded soundly to a particular wavelength at which *E*_g_ ≈ 3.5 eV, and the band gap was attained through optical analysis (Fig. 1(c)). However, further increment in the scanned wavelength (*λ* ≥ 720 nm) brought about a slight figure of merit enhancement with a value of 11.9 mA W^−1^. This is mainly because of the window effect of the Si wafer as the wavelength passes into/near the IR region.^3^ The D* and LDR are shown in the ESI (Fig. S2†) as the wavelength-dependent figure of merits, in which the addressed figure of merits exhibited a similar response profile to those outlined in Fig. 4(b and c).

#### 3.2.3 Incident power profile

**Fig. 5:**
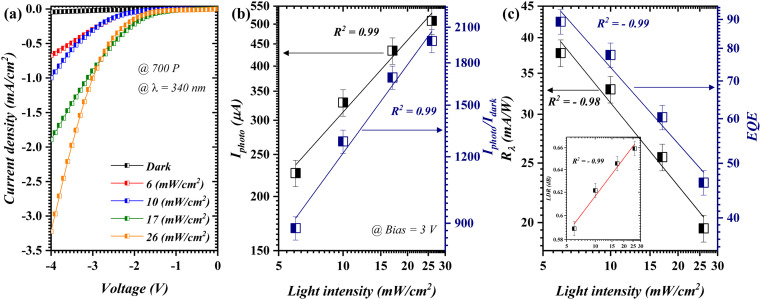
Opto-electrical features of the fabricated photodetector at 700 pulses as a function of incident power; (a) *I*–*V* profile, (b) *I*_photo_ and *I*_photo_/*I*_dark_ ratio, and (c) *R*_*λ*_ and EQE.

Fig. 5 depicts the opto-electrical characteristics as a function of the incident power for the photodetector fabricated using 700 pulses. The utilized wavelength in this investigation (*λ* = 340 nm) is the closest value to the cut-off wavelength demonstrated in the optical analysis (*λ*_C_ = 335 nm). The total induced current (Fig. 5(a and b) – black line) increased progressively as the incident power was increased from 6 to 26 mW cm^−2^. This can be justified by means of the photo-excited carrier within the heterojunction framework.^30^ The *I*_photo_/*I*_dark_ ratio (Fig. 5(b)) revealed the linear dependency along the applied *P*_in_ with a ratio as high as 1979%. Fig. 5(c) outlines the *R*_*λ*_ (black line) and EQE (navy line), in which the *R*_*λ*_ and EQE exceeded 37.8 mA W^−1^ and 137.7, respectively, at an incident power as low as 6 mW cm^−2^. Theoretically, the value of *R*_*λ*_ increases as the incident power decreases until it reaches a saturation value, as these parameters are inversely proportional (*P* ∝ *R*_*λ*_^−1^). Accordingly, if the dark current (25 μA@3 V bias) was attained at an incident power of 1 mW cm−2, *R*_*λ*_ would unquestionably surpass a value of 25 mA W^−1^. This was further articulated through the LDR profile, as shown in the inset of Fig. 5(c). Specifically, the LDR results suggest a clear positive linear proportionality to the optical signal input. The D* and LDR are shown in the ESI (Fig. S3†) as the incident power dependence figure of merits.

#### 3.2.4 Switching behavior profile – function of incident power and wavelength

**Fig. 6:**
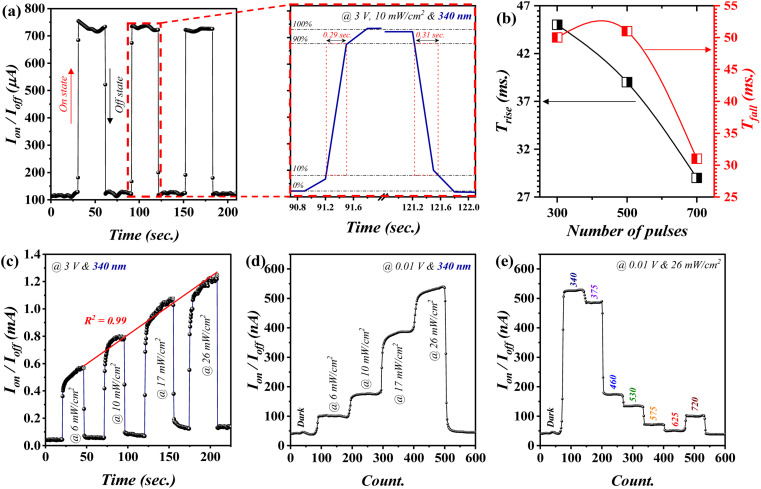
Time-resolved characterization for the optimum device (at 700): (a) switching behavior, (b) rise/fall time as a function of the utilized laser pulses, (c) switching behavior with a bias of 3 V, the optimum device (@700) performance at extremely low voltage (0.01 V) as a function of (d) incident power and (e) wavelength profiles.

Fig. 6 presents a complete time-resolve characterization of the fabricated photodetectors (@700). Fig. S4 (ESI†) shows the characterization of the devices fabricated using 300 and 500 laser pulses. The switching characteristics (Fig. 6(a)) exhibited a rapid photocurrent increment upon 340 nm UV illumination from 100 μA (OFF state) up to a stable value at around 750 μA (ON state). The described behavior was periodically repeated over three cycles with a pulse width of ∼31 s, which indicates the functional stability and robustness of the proposed device. The discussed photodetector exhibited considerably rapid rise/fall periods of 0.29 and 0.31 s, respectively, as shown in the inset of Fig. 6(a). The investigated rise/fall time showed a decreasing trend with increasing laser pulses (Fig. 6(b)). However, even for the device fabricated using 300 pulses, the time-resolved characterization delivered a noticeably rapid profile. In comparison to devices fabricated using 300 and 500 pulses, the photodetector attained using 700 pulses demonstrated a faster time-resolved property, which could be due to the relatively smoother photo-excited electron transfer pathway.^31^ Furthermore, the incident power dependency of the examined time-resolved characteristic was tested continuously with power alteration (Fig. 6(c)). The presented outcome suggested a positive linear incident power dependency with a *R*^2^ value of 0.99. The optimum device (@700) demonstrated, to a certain extent, a self-driven feature for which the indicated device exhibited a noticeable *I*_photo_ with increasing incident power (Fig. 6(d)). This was acquired at a very small bias (0.01 V). Such profile was also perceived by means of wavelength at the pronounced bias. In particular, the *I*_photo_ value of ∼525 nA was attained at a wavelength of 340 nm, after which relatively low *I*_photo_ values were attained at higher wavelengths (Fig. 6(e)). This suggests the ability of the proposed geometry to be considered as a self-biased visible blind photodetector.

Table 1 represents a comparison of the attained figure of merits for the proposed geometry, along with other reported studies concerning the fabrication of the SnO_2_/Si heterojunction photodetector.

**Table 1:** Proposed geometry with other reported studies in the UV region

Materials	*R*_*λ*_ (A W^−1^)	*I*_photo_/*I*_dark_	D* (jones)	*T*_rise/fall_ (s)	Ref.
SnO_2_/Si	0.512	—	—	0.018/0.029	18
SnO_2_/Si	0.062	1.8	—	—	19
SnO_2_/Si	0.66	9.33	1.38 × 10^11^	—	32
SnO_2_/Si	0.033	12.82	1 × 10^15^	0.29/0.31	This study

## 4. Conclusion

A nanostructured SnO_2_/Si heterojunction UV photodetector was successfully fabricated as a function of laser pulses using the PLD approach. The attained outcomes indicated that an augmentation in the pulses resulted in a preferable photodetection mechanism effect. The *R*_*λ*_ increased from 22.6 to 32.9 mA W^−1^ using 300 and 700 pulses, respectively, at an incident power of 10 mW cm^−2^. A robust correlation between the optical analysis of the deposited SnO_2_ layer and the photodetector spectral response characteristics was also demonstrated. In detail, the device attained at 700 pulses (10 mW cm^−2^) demonstrated a decreasing *I*_photo_ behavior from 330 to 63.9 μA at wavelengths of 340 and 625 nm, respectively, which shows the ability of the proposed geometry of visible light rejection. The incident power profile revealed the *R*_*λ*_ value dependency for the optimum photodetector from 6 to 26 mW cm^−2^. The indicated device exhibited a substantial response/recovery time that was estimated to be 0.29 and 0.31 s, respectively. The proposed work elucidates a straightforward approach for a visible blind, fast-response and self-driven optoelectronic design.

## Data availability

Data are available upon request from the authors.

## Conflicts of interest

The author declares no conflict of interest.

## Supplementary Material

RA-014-D4RA05303F-s001
