# Supplementary material for: Opto-electrical evaluation of visible blind fast-response nanostructured SnO2/Si photodetector
Source: RSC Adv. 2024 Sep 2;14(38):27733–40. doi: 10.1039/d4ra05303f (PMC11367619; doi:10.1039/d4ra05303f)
Supplement: RA-014-D4RA05303F-s001 [file RA-014-D4RA05303F-s001.pdf]

Supplementary Information (SI) for RSC Advances.
This journal is © The Royal Society of Chemistry 2024

Supplementary Materials

Opto-electrical evaluation of visible blind fast-response nanostructured SnO_2/Si photodetector

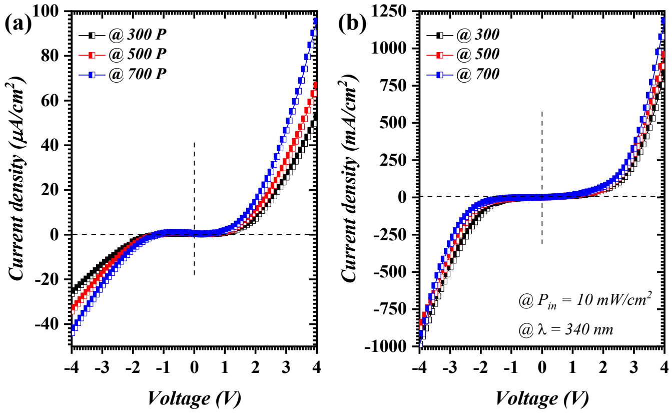

Figure *S1*: Opto-electrical features of the proposed devices as by means of laser pulses: (a) dark I-V and (b) illuminated I-V @ $340\ nm$, and $10\ mW/cm^2$.

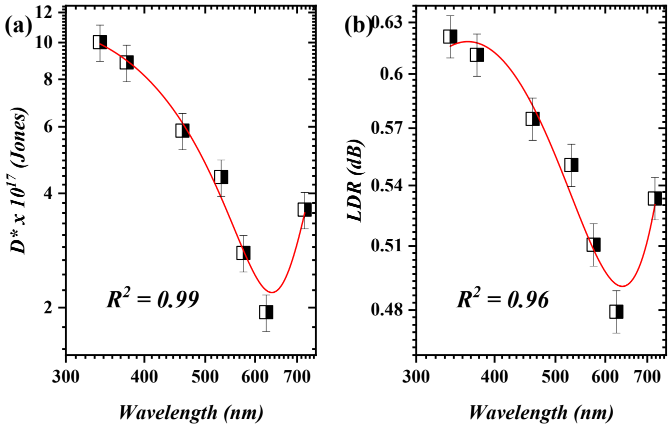

Figure *S2*: Opto-electrical features of the fabricated photodetector @ 700 pulses as a function of wavelength; (a) photo-detectivity and (b) linear dynamic range characteristics were attained @ $3\ V$ and $10\ mW/cm^2$.

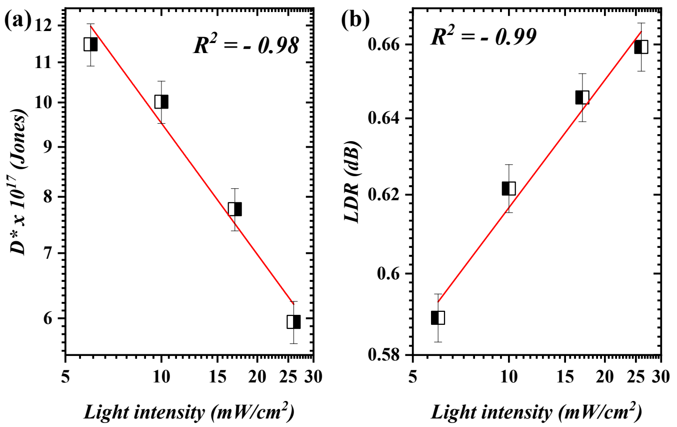

Figure ***S3***: Opto-electrical features of the fabricated photodetector @ 700 pulses as a function of illumination power; (a) photo-detectivity and (b) linear dynamic range characteristics were attained @ $3\ V$ and $\lambda = 340\ nm$.

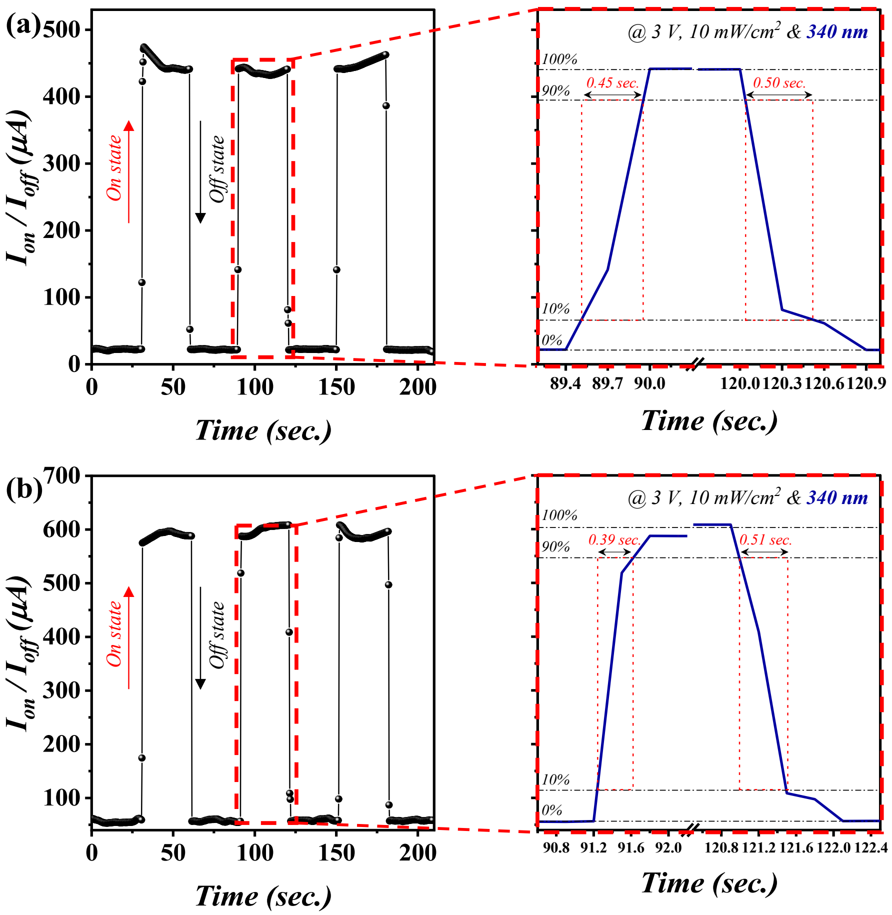

Figure ***S4***: Time-resolved characteristics of the fabricated devices at (a) 300 pulses and (b) 500 pulses.
